# Sustained Exposure of Substance P Causes Tendinopathy

**DOI:** 10.3390/ijms21228633

**Published:** 2020-11-16

**Authors:** Seo Yoon Oh, Do Kyung Kim, Soo Hong Han, Hyun Hae Lee, Yunhui Jeong, Minjung Baek, Hyeongkyung Kim, Wooyeol Ahn, Soonchul Lee

**Affiliations:** 1 Department of Molecular and Cell Biology, University of California, Berkeley, CA 94720, USA; seoyoonoh@berkeley.edu; 2 CHA Graduate School of Medicine, 120 Hyeryong-ro, Pocheon 11160, Korea; dokim1018@chauniv.ac.kr; 3 Department of Orthopaedic Surgery, CHA Bundang Medical Center, CHA University School of Medicine, Seong-nam 13496, Korea; hsoohong@cha.ac.kr (S.H.H.); aotcnlsl@gmail.com (H.H.L.); jeongyunhui92@gmail.com (Y.J.); eclsa79@gmail.com (M.B.); jongbumoh@gmail.com (W.A.); 4 Department of Pathology, Kyung Hee University Hospital at Gangdong, Kyung Hee University College of Medicine, Seoul 05278, Korea; shbyun@khu.ac.kr

**Keywords:** substance P, tenocyte, tendinopathy, degenerative, tendon

## Abstract

Recently, neuromediators such as substance P (SP) have been found to be important factors in tendon homeostasis. Some studies have found SP to be the cause of inflammation and tendinopathy, whereas others have determined it to be a critical component of tendon healing. As demonstrated by these conflicting findings, the effects of SP on tendinopathy remain unclear. In this study, we hypothesized that the duration of SP exposure determines its effect on the tendons, with repetitive long-term exposure leading to the development of tendinopathy. First, we verified the changes in gene and protein expression using in vitro tenocytes with 10-day exposure to SP. SP and SP + Run groups were injected with SP in their Achilles tendon every other day for 14 days. Achilles tendons were then harvested for biomechanical testing and histological processing. Notably, tendinopathic changes with decreased tensile strength, as observed in the Positive Control, were observed in the Achilles in the SP group compared to the Negative Control. Subsequent histological analysis, including Alcian blue staining, also revealed alterations in the Achilles tendon, which were generally consistent with the findings of tendinopathy in SP and SP + Run groups. Immunohistochemical analysis revealed increased expression of SP in the SP group, similar to the Positive Control. In general, the SP + Run group showed worse tendinopathic changes. These results suggest that sustained exposure to SP may be involved in the development of tendinopathy. Future research on inhibiting SP is warranted to target SP in the treatment of tendinopathy and may be beneficial to patients with tendinopathy.

## 1. Introduction

Tendinopathy is the most common tendon disorder, and its incidence continues to rise due to increasing involvement of humans in recreational sports [1,2,3]. As a chronic musculoskeletal disorder, tendinopathy results in the degeneration of collagen protein in the tendons, most commonly in the Achilles, patellar, rotator cuff, and forearm extensor tendons. However, this clinical problem can occur in any tendon as a consequence of increased activity, weight, age, or genetic factors, and it is often observed near an area of concentration of tendons or in sites where tendons or ligaments are inserted into the bone [1,4,5]. In addition to tendinosis, this disorder is strongly associated with activity-related pain, focal tendon tenderness, and decreased strength and movement in the affected area [2,3,6,7]. Cells present in the tendinopathic tendons are known to exhibit several distinct features. They are rounder and higher in number than in healthy tendons, and they manifest signs of oxidative damage and apoptosis [1,8,9]. Furthermore, they produce a matrix with decreased collagen type 1 and are less mature. As this normal cell matrix complex is altered, “relative” stress deprivation occurs [10,11]. The metalloproteinase matrix destruction begins, which induces pain in the enthesis [1]. According to data retrieved from histological studies, vascular and neuronal infiltration subsequently occur to repair the damaged area [12].

Although it is accepted that the main cause of tendinopathy is the buildup of minor injuries that have not healed sufficiently, the underlying biological mechanism and advancement of disease is not clear [2]. Recently, neuromediators such as substance P (SP) have been found to be critical factors in tendon homeostasis. Disruption of its regulation may heavily contribute to the progression of neurogenic inflammation and tendinopathy [13,14,15,16]. However, other studies state the contrary that SP accounts for a critical component of tendon healing [17,18]. Studies focusing on the effects of SP on the tendons show that SP enhances the proliferation of pluripotent tendon cells and accelerates angiogenesis and hypercellularity in the tendon tissue [19,20]. SP administered to rat tendons has also demonstrated that this neuropeptide enhances healing by promoting early tissue proliferation and regulating sensory nerve ingrowth [17]. As demonstrated by these conflicting hypotheses, the effects of SP on tendinopathy remain unclear. Furthermore, SP is implicated in tendinopathy development but its specific role as a causative agent or a resulting factor of tendinopathy has not been determined. In this study, we hypothesized that SP may be the cause of tendinopathy. In addition, we focused on SP exposure duration on the tendons to demonstrate that repetitive long-term exposure causes tendinopathy.

## 2. Results

### 2.1. In Vitro Experiments

To evaluate the proliferation of human tenocytes, three different experimental groups with different SP treatments on human tenocytes were evaluated on days 0, 2, 6, and 10. Control: phosphate-buffered saline injection (no SP treatment), Once: one-time SP treatment on day 0, Daily: daily SP treatment. All three experimental groups showed a steady increase in cell proliferation rate at all time points. Among the experimental groups, the Daily group showed the highest cell proliferation. Cell proliferation was evaluated in triplicate.

#### 2.1.1. SP Increased Tenocyte Proliferation

**Figure 1:**
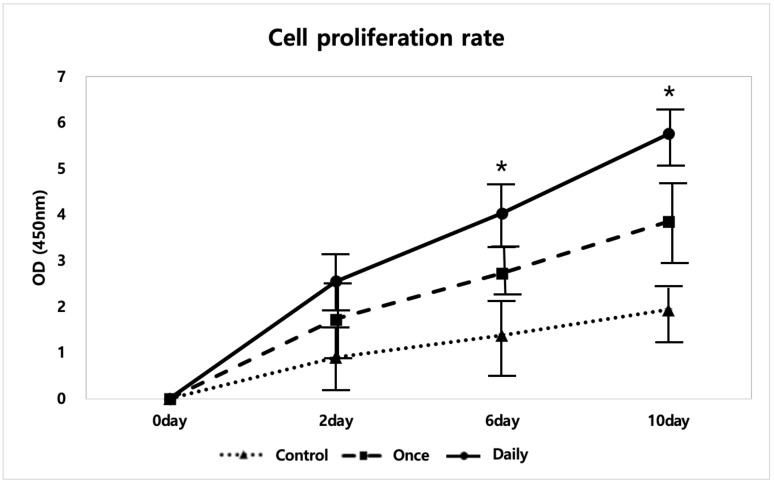
Cell proliferation evaluation of tenocytes at different time points of substance P (SP) treatment. Data are represented as mean ± standard deviation. Kruskal-Wallis test with Tukey’s post-hoc test was used for statistical evaluation. *n* = 3. * *p* < 0.05.

As seen in Figure 1, both the Once and Daily groups had increased proliferation rates after SP injection compared to the Control group. As the number of days in culture progressed, all three groups, the Control, Once, and Daily groups, showed a steady increase in proliferation. However, the Once and Daily groups displayed a steeper increase. The Daily group had a significantly higher cell proliferation rate after 10 days of culture (*p* < 0.05; Figure 1).

#### 2.1.2. Continuous SP Exposure Induced Inflammation and Deteriorates Collagen Synthesis In Vitro

**Figure 2:**
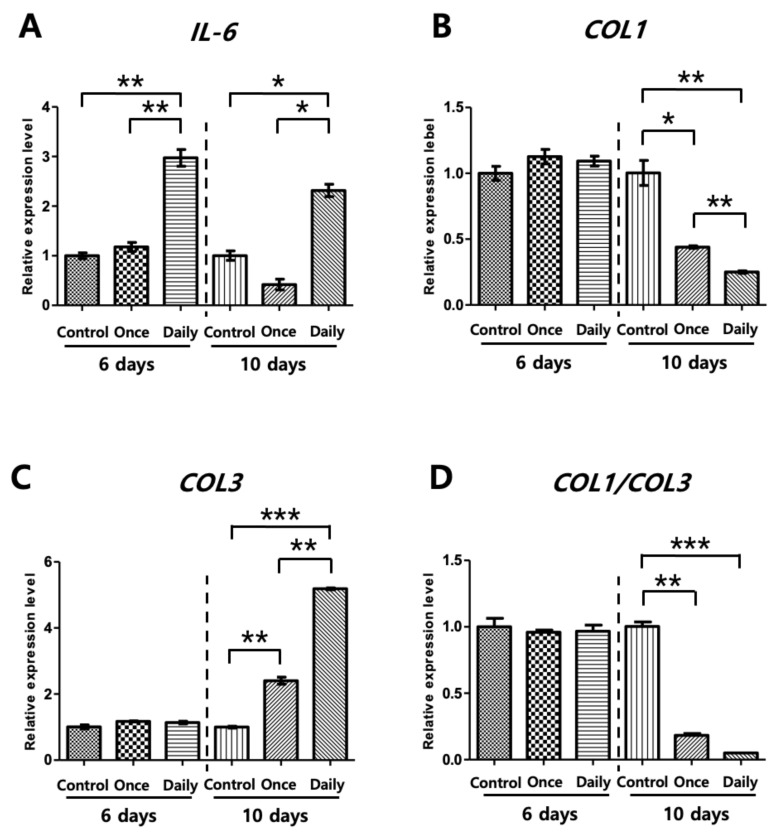
Quantitative real-time polymerase chain reaction (qRT-PCR) evaluation at 6 and 10 days of cell culture. To evaluate the inflammatory response and gene expression of collagen type 1 (*Col1*) and collagen type 3 (*Col3*), quantitative real-time polymerase chain reaction (qRT-PCR) was conducted on day 6 and day 10 of cell culture. The Daily group showed a significant increase in *IL-6* expression, a proinflammatory cytokine gene. (**A**) *Col3* expression was increased by substance P (SP); however, expression of *Col1* was decreased 10 days after Daily SP treatment. The *Col1*/*Col3* ratio decreased significantly in all experimental groups (**B**–**D**). Experiments were performed in triplicate. Control: phosphate-buffered saline injection (no SP treatment), Once: one-time SP treatment on day 0, Daily: daily SP treatment. Data are represented as mean ± standard deviation. Kruskal-Wallis test with Tukey’s post-hoc test was used for statistical evaluation. *n* = 5. * *p* < 0.05, ** *p* < 0.01, *** *p* < 0.001.

Total RNA was extracted on 6 days and 10 days from cultured human tenocytes treated with phosphate-buffered saline (PBS) or SP. Quantitative real-time polymerase chain reaction (qRT-PCR) analysis was performed to examine the changes in expression levels among the inflammatory response and collagen deposition genes such as *IL-6*, *Col1*, and *Col3*. There was a significant increase in *IL-6* in the Daily group. In addition, the injection of SP induced an increase in *Col3*, a gene linked to immature and disorganized collagen type with structural weakness in the tendons. Simultaneously, there was a decrease in *Col1* at the end of 10 days. Therefore, the ratio of *Col1*/*Col3* decreased significantly in the Daily group. The *Col1* gene is abundantly expressed in bones and normal collagen [21]. During the regenerative phase, *Col1* is abundant; however, an increase in *Col3* instead results in microtears, and the buildup of microtears progresses into tendinopathy (Figure 2) [1,22].

**Figure 3:**
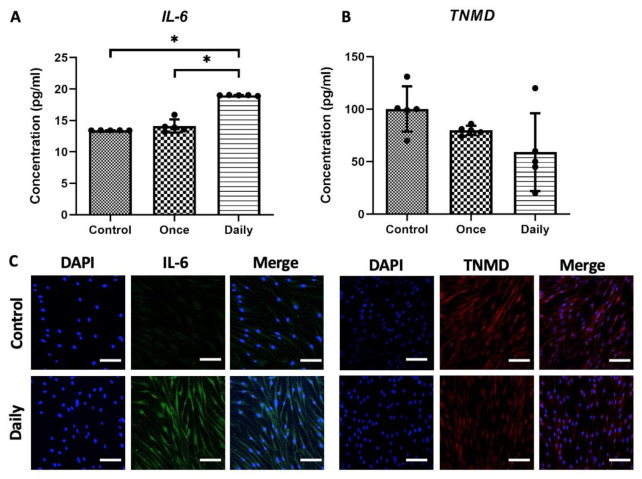
Enzyme-linked immunosorbent assay (ELISA) results of proinflammatory marker and tendon-specific marker in tenocytes treated with phosphate-buffered saline or substance P (SP). Interleukin-6 (IL-6) concentration in growth media on day 10, measured using ELISA. Data are represented as mean ± standard deviation. Kruskal-Wallis test with Tukey’s post-hoc test was used for statistical evaluation. *n* = 5. * *p* < 0.05. (**A**). Tenomodulin (TNMD) protein concentration in growth media on day 10, measured using ELISA. Scattered dots are used to show variables. (**B**). Cells were counterstained with DAPI (4′,6-diamidino-2-phenylindole, dihydrochloride) to show nuclei (blue), *IL-6* (green), and expression of TNMD (red) (**C**). Original magnification: 200×. Experiments were performed in triplicates. Scale bar = 50 µm.

Next, the *IL-6* and tenomodulin (TNMD) protein concentrations in growth media at 10 days of cell culture were measured using enzyme-linked immunosorbent assay (ELISA). The *IL-6* protein level showed a significant increase at 10 days in the Daily group. However, there was no statistically significant difference in the level of the tenocyte marker, TNMD, among the Control, Once, and Daily groups (Figure 3A,B).

Immunocytochemistry (ICC) evaluation of tenocytes showed a similar trend. Tenocytes in the Daily group were readily identifiable for *IL-6* and had lower signal intensity of TNMD. Most cells expressed *IL-6* in the Daily group compared to the Control group at 10 days of cell culture. Morphological differences were observed between the Control and Daily groups. The morphology of tenocytes stained with TNMD in the Control group showed a spindle-like shape, producing a parallel array (Figure 3C).

### 2.2. In Vivo Experiments

#### 2.2.1. SP Injection Deteriorated the Tendon Strength

**Figure 4:**
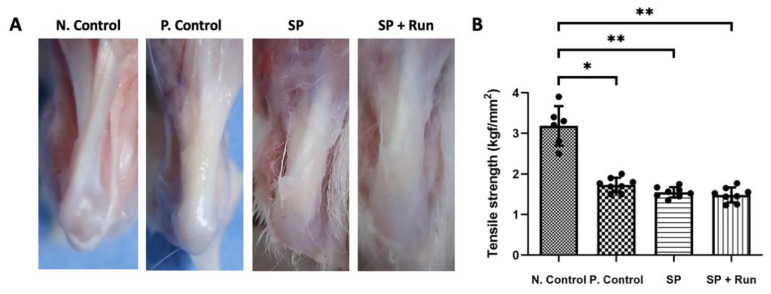
Gross phenotypes of the Achilles tendons and tensile strength of muscle-tendon-bone complex. The Negative (N.) Control tendon had a shiny smooth gross appearance in comparison to the others. However, the Positive (P.) Control, SP, and SP + Run groups showed dull and thicker tendons with a yellowish hue. In particular, SP + Run group showed the most severe change (**A**). Biomechanical testing was used to compare the tensile strength among Control, SP with treadmill running, SP, and SP + Run groups. Both SP and SP + Run groups had significantly lower tensile strength compared to the N. Control, similar to the P. Control. Data are represented as mean ± standard deviation. Kruskal–Wallis test with Tukey’s post-hoc test was used for statistical evaluation. *n* = 6 (N. control), *n* = 8 (P. control, SP, SP + Run). * *p* < 0.05 and ** *p* < 0.01. Scattered dots are used to show variables. (**B**).

After 2 weeks of SP injection, the gross appearance of tendon specimens was observed to determine the effects of SP injection and SP injection with treadmill running. The harvested Negative (N.) Control tendon showed an apparent normal tendon structure without any swelling or inflammation. Compared with the N. Control, P. Control, SP, and SP + Run groups showed yellowish, thickened tendons, which points to swollen tendons due to inflammation. Among the experimental groups, the SP + Run group showed the most severe appearance with a dull yellowish thickened tendon (Figure 4A).

Biomechanical testing revealed that the N. Control group had a significantly higher (1.88 times) tensile strength than the other groups (*p* < 0.05 or 0.01). However, there were no significant differences between the P. Control, SP, and SP + Run groups (Figure 4B).

#### 2.2.2. Multiple SP Injections Induced Histological Tendinopathic Changes

**Figure 5:**
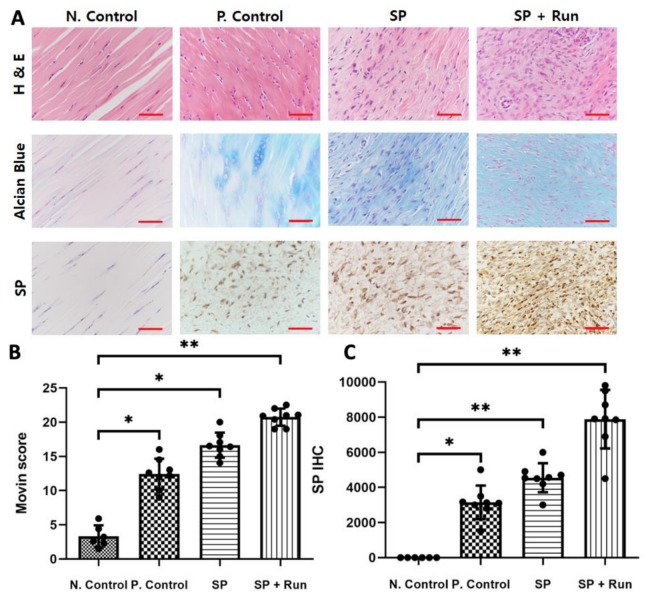
Histopathological changes and quantification after multiple substance P (SP) administration. Hematoxylin and eosin (H and E) and Alcian blue staining were used for the observation of SP and the appearance of collagen fibers and tenocytes (**A**,**B**). Movin scores used for selected samples to quantify the amount of tendon degeneration shown using H and E and Alcian blue staining (**C**). Data are represented as mean ± standard deviation. Kruskal-Wallis test with Tukey’s post-hoc test was used for statistical evaluation. *n* = 6 (N. control), *n* = 8 (P. control, SP, SP + Run). * *p* < 0.05 and ** *p* < 0.01. Scattered dots are used to show variables.

Hematoxylin and eosin (H and E) staining of the N. Control tendons displayed a uniform appearance of compact, well-aligned collagen fibers with normal spindle-shaped tenocytes disposed parallel to the fiber pattern. However, tendinopathic changes were observed in the P. Control group. More severe changes were observed in SP and SP + Run groups with disorganized collagen bundles, a loss of polarity, and increased cell number. Alcian blue staining also showed that the SP and SP + Run tendons had an increased ground substance consisting of proteoglycans and glycosaminoglycans with several mucoid patches and vacuoles between fibers. Further, SP + Run group had the highest Movin score, which indicated the most severe degenerative change (Figure 5A,B).

Additionally, SP ICC analysis demonstrated increased staining density in P. Control, SP, and SP + Run groups in comparison to the N. Control samples. SP was barely observed in the N. Control, but the P. Control, SP, and SP + Run groups displayed positive staining for the SP protein. SP was especially strongly represented in the SP + Run group. Representative histological sections of the longitudinal axis of the central region of the tendon insertion are shown in Figure 5A,C.

## 3. Discussion

SP is implicated in tendinopathy development; however, whether it is the cause or result remains unclear. Using in vitro and in vivo experiments, we tested our hypothesis that the duration of SP exposure determines its effect on the tendons. While brief exposure to SP is closely related to tendon healing, repetitive long-term exposure is strongly linked to the development of tendinopathy [22,23]. Our quantification using different histological factors and analysis of varying tendon strengths after injection of SP revealed that SP is a cause of tendinopathy.

Past studies have focused on various methods to understand the underlying biological mechanisms and advancement of tendinopathy. Some have examined the integrity of the tendon matrix and biomolecular changes to explain how tendinopathy is induced when tendon cells are subjected to repetitive loads. Other studies have emphasized the connection between SP and enhanced cellular proliferation [1,24]. Zhou et al. demonstrated how SP induces tendinopathy in a dose-dependent manner. Their conclusion that tendinopathy is induced with increased dosage corroborates our results. An increase in dosage translates to a longer half-life and thus a longer duration of SP exposure in the tendons, resulting in tendinopathy. With knowledge from previous research that SP is involved in chronic inflammation, neuropathic pain, and tendinopathy and is critical for cell proliferation, we aimed to show the varying effects of SP depending on its duration of exposure on the tendons in vitro and in vivo [25,26,27]. Barbe et al. reported that SP plays a crucial role in the fibrogenic responses and proposed blocking SP-neurokinin-1 receptor signaling with a neurokinin 1 receptor antagonist as a therapeutic target for musculotendinous and dermal fibrosis [28].

Fedorczyk et al. reported that exposure to SP, IL-1β, and connective tissue growth factor showed exposure-dependent changes in tendinosis in flexor digitorum tendons of rats [29].

Analyzing the optical density (OD) levels among the three groups, the Control, Once, and Daily, in the in vitro experiments showed an increase in proliferation rates after SP injection, with the Once and Daily groups displaying significantly increased OD levels compared to the Control. Such data allowed us to deduce that SP increased tenocyte proliferation. The inflammatory responses of SP were also analyzed according to the duration of exposure. Continuous SP exposure induced inflammation and deteriorated collagen synthesis in vitro, which was validated by gene analysis used to identify changes in expression levels of *IL-6*, *Col1*, and *Col3*. *IL-6*, the genes related to inflammatory response, showed a significant increase. *Col3* is immature collagen that increases during the proliferation phase or damage. *Col1* is found in the mature tendon matrix or during the maturation stage of the tendon remodeling process. During tendon remodeling, collagen fibers start to organize along the longitudinal axis of the tendon, thereby restoring tendon stiffness and tensile strength by replacing *Col3* with *Col1* [30,31]. However, compared with native tendon, repaired tendon was reported to show decreased mechanical strength by decreased collagen fiber integration with higher ratio of *Col3* to *Col1* [31]. The ratio of *Col1*/*Col3* decreased in the Daily group. Furthermore, ELISA showed that proinflammatory cytokine levels increased in growth media. This result was further confirmed using *IL-6* and TNMD staining, in which an increase in *IL-6* and a decrease in TNMD, a glycoprotein required for tendon maturation and tenocyte proliferation, was observed in the Daily group compared to the Control group.

Our in vivo experiments that included biomechanical testing revealed a deterioration of tendon strength after SP injection. Histopathological analysis using H and E and Alcian blue staining revealed that the SP + Run group had the most severe degenerative changes with the highest Movin score and immunohistochemically clear representation of SP.

One of the most evident strengths of our study is its potential to be applied to creating animal models. To the best of our knowledge, there are two popular animal models to investigate tendinopathy. First, collagenase could be injected into the tendon to create tendinopathy, but it was reported that this model is closer to the acute injury model, not chronic tendinopathy [32,33]. Furthermore, past studies have used animals running on treadmills as their models in the generation and observation of tendinopathy. However, this model is time consuming and requires a treadmill machine specific for rodents. On the contrary, our model that includes the SP injection in normal tendons results in tendinopathy development within just 2 weeks. Compared to the 7 weeks required for past treadmill running models, our model is useful and efficient. In a prospective manner, according to the results of this study, clinical medication inhibiting SP and reducing overuse of tendons may be beneficial in the management of patients with tendinopathy.

The limitations of this study are that our findings of SP in tendinopathy are confirmed only in animal models. Previous studies have shown that exposure to SP in a dose- and time-dependent manner causes changes in the expression of connective tissue growth factor and fibrogenic process of tenocytes [34]. In this study, the dosage and duration of SP exposure was not optimized. However, we were able to make our selection by performing a preliminary study that tested two different dosages of SP (10^−5^ M and 10^−4^ M; unpublished data). In addition, we administered SP via an injection, but SP overexpression through genetic modification may also be a potential area of interest for further research. A previous study reported difficulties in forcing rodents to run to induce pathological changes as wild rodents are known to run up to 15 km/day [35]. The treadmill study we conducted had rats run at a speed of 17 m/min, 1 h per day. This time duration and speed may be insufficient to generate pathological changes. However, our study aimed to evaluate the effect of SP on tendinopathy using a treadmill. To investigate the effects of different treadmill exercise durations and speeds in relation to changes in tendinopathy, a treadmill study with different settings of time and speeds should be performed.

## 4. Materials and Methods

### 4.1. Cell Culture

The human tenocytes were cultured in Dulbecco’s modified Eagle’s medium supplemented with 10% (*v*/*v*) fetal bovine serum and 1% (*v*/*v*) penicillin-streptomycin. Human tenocytes were purchased from Zen-Bio (NC, USA). The cells were divided into three groups based on the duration of SP exposure: Control (no SP treatment), Once (10^−5^ M SP once on day 0), and Daily (10^−5^ M SP daily for 10 days). For the Control and Once groups, the same dose of PBS was used as Control when SP was not administered. After 10 days of cell culture, cell viability was measured using the WST-1 assay. Next, qRT-PCR and ICC were used to analyze the tendinopathy-related markers to determine the different effects of the duration of SP treatment.

### 4.2. Cell Viability Test

Cell viability was measured using the WST-1 assay. The tenocytes were seeded into 96-well plates at a density of 2 × 10^3^ cells/well and treated with SP for 0 (baseline), 2, 6, and 10 days. After incubation, 10 µL of the WST-1 solution (cat. no. W201, Dojindo Molecular Technologies, MD, USA) was added to each well, and the plates were incubated for 3 h at 37 °C in 5% CO_2_. The optical densities were measured at 450 nm using EON Gen software version 5.2 (BioTek Instruments, VT, USA). Readings were taken at least thrice at all time points for each sample.

### 4.3. RNA Isolation and qRT-PCR

Total mRNA was isolated from the cultured tenocytes and homogenized using TRIzol reagent (Invitrogen, MA, USA) according to the manufacturer’s instructions. A total of 1 µg RNA from each sample was reverse-transcribed into complementary DNA (cDNA) using an iScript cDNA Synthesis Kit (cat. no. 1708890, Bio-Rad Laboratories GmbH, Feldkirchen, Germany). PCR was performed in triplicate using Bio-Rad CFX96 Touch Real-Time PCR Detection System. Amplification of total RNA was performed using the TaqMan Gene Expression Assay (Applied Biosystems, Warrington, UK) for the analysis of *IL-6* (Hs00174131_m1), *Col1* (Hs00164004_m1), and *Col3* (Hs00943809_m1). Transcript levels were normalized against glyceraldehyde 3-phosphate dehydrogenase gene expression (ABI code: Hs99999905_m1), which was expressed stably across the sample, and gene expression was calculated using 2^−∆∆Ct^ method. For a more accurate analysis, transcript levels were measured at least thrice for each sample.

### 4.4. ELISA of IL-6 and TNMD

ELISA was performed using the Quantikine Immunoassay ELISA Kit for human *IL-6* (cat. no. D6050; R & D Systems, MN, USA). The ELISA kit for human TNMD (cat. no. CSB-EL024007HU; Cusabio, Wuhan, China) was also used. All assays were performed according to the manufacturer’s recommendations.

### 4.5. ICC of Tenocytes

During cell culture, we confirmed the identity of tenocytes using ICC with 2 × 10^3^ cells. After transferring 200 µL of cell culture to the 8-well chamber slide, the cells were grown to confluence with the addition of fresh media for 1 day. After washing with PBS, the cells were fixed using 4% formaldehyde. Permeabilization of the membranes was performed by incubating the slides with 0.25–0.5% Triton X-100 in PBS for 10 min, followed by blocking with 1% bovine serum albumin for *IL-6* and TNMD, respectively. The cells were incubated with mouse anti-IL-6 monoclonal antibody (1:200 dilution; Santa Cruz Biotechnology, CA, USA; code: sc-130326) and goat anti-TNMD antibody (1:50 dilution; Abcam, MA, USA; code: ab203676) overnight at 4 °C. After additional washing, the secondary antibodies were incubated in the dark for 30 min at room temperature and mounted in a VECTASHIELD Antifade Mounting Medium (H-1000-10) for fluorescence (Vector Laboratories, CA, USA). An Olympus CKX41 microscope equipped with epifluorescence and an Olympus XC10 digital camera were used for the analysis.

### 4.6. In Vivo Experiment Design

This study was approved by the University of CHA Animal Care and Use Committee. The ethical approval code (IACUC170082) was given by University of CHA Animal Care Center. Thirty Sprague-Dawley rats were used; the rats were divided into four groups consisting of healthy controls (N. Control, *n* = 6), running only (P. Control, *n* = 8), SP injection only (SP, *n* = 8), and SP injection + treadmill running (SP + Run, *n* = 8). Table 1 presents the animal study design. The animals were made to run on the treadmill or have either PBS or SP injected into their Achilles tendon. Rats in the N. Control, SP, and SP + Run groups were sacrificed at 2 weeks after their first injection. The rats in the treadmill running group were sacrificed after a 7-week running program according to a previously established protocol [33].

### 4.7. Injection of SP and Treadmill Running

**Figure 6:**
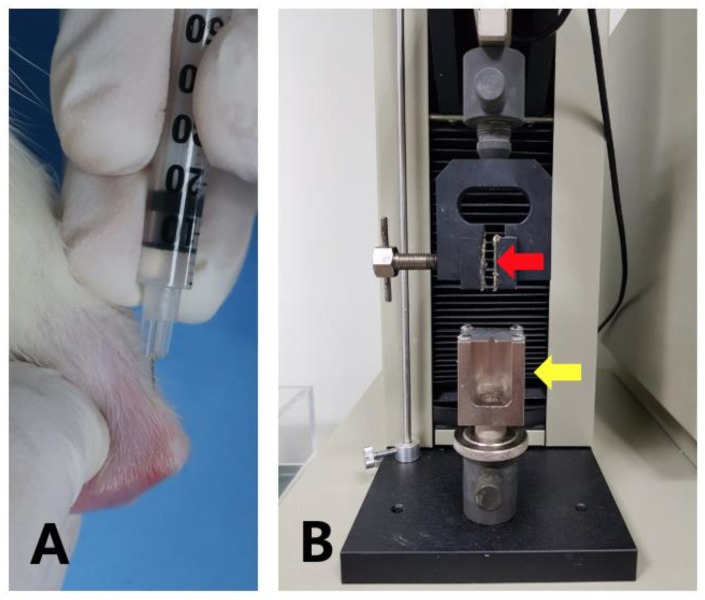
Injection in the Achilles tendon, treadmill running, biomechanical test machine. Using a 27-G needle, phosphate-buffered saline or substance P (SP) was injected to the insertion of the Achilles tendon. To make the needle direction parallel to the long axis of the Achilles tendon, the needle was bent before injection. After the Achilles tendon was fully stretched through knee extension and ankle dorsiflexion, the needle was introduced distally from the musculotendinous junction (**A**). A biomechanical test machine was used to measure the tensile strength of the muscle-tendon-bone complex. Red and yellow arrows indicate sample holders for the musculotendinous junction and bone tendon complex, respectively (**B**).

The rats were anesthetized with isoflurane in an anesthetic induction chamber and then in a mask. SP and SP + Run groups were administered 20 µL of SP (10^−8^ µmol/Kg), while the N. Control group was administered the same quantity of PBS on the insertion area of the Achilles tendon using a 27-G needle every other day for 2 weeks. To make the needle direction parallel to the long axis of the Achilles tendon, the needle was bent before injection. After the Achilles tendon was fully stretched through knee extension and ankle dorsiflexion, the needle was introduced distally from the musculotendinous junction (Figure 6A).

Rats in the P. Control group were subjected to running on the treadmill machine at 17 m/min, 0° slope, 1 h/day, 5 days/week for a total of 7 weeks. For effective running and adaptation, rats were trained for 1 week with a gradual increase in intensity.

### 4.8. Mechanical Test of Harvested Tendons

A mechanical test was conducted using a universal testing machine. The protocol was consistent for all samples: dissection of the tendon from muscle tissue, keeping the patella in the upper side and the tibial bone insertion on the lower side. The tendon was gripped with an innovative clamping system. The mechanical properties of the tendon tissues were tested between two grips. The gastrocnemius-soleus muscles were clamped between two pieces of sandpaper in a tissue grip with serpentine surfaces designed to prevent slippage. The Achilles tendon-calcaneal junction was placed in the slit of a cylindrical clamp, which was tightened to prevent the calcaneus from pulling out. In the central area of tendon, two marks were created at a distance of 4 mm from the clamps using black ink. In this experiment, all the tests were performed in displacement control mode to check actuator movement through parameters such as maximum and minimum values and ramp rate were expressed in mm and mm/s, respectively. Load and displacement data were saved in a comma-separated value format. The image sequences were acquired during the tests using a custom interface implemented in the universal testing machine program (MC-Tester). Tendon specimens were excluded from analysis if they slipped or failed during the mechanical test (Figure 6B).

### 4.9. Histology

After euthanization, the Achilles tendon of each rat was dissected carefully from the attached calcaneal bone. The samples were fixed using 4% buffered formalin, embedded in paraffin, and stained with H and E and Alcian blue to quantify the severity of tendinopathy. Tissue changes were evaluated by histological criteria according to the Movin score. Images were obtained using a high-resolution Leica DC200 digital imaging system (Leica Microsystems, Wetzlar, Germany) mounted on an Olympus DMLB microscope. The Movin score was used to quantify the amount of tendon degeneration using H and E and Alcian blue staining (Supplementary Table S1) [36,37]. Immunohistochemistry with SP antibody (ab14184, Abcam, Cambridge, MA, USA) was performed on 5-μm-thick formalin-fixed, paraffin-embedded blocks on a Bond-III Automated IHC Stainer (Leica Biosystems). The SP antibody was diluted (1:1000) and the BOND Polymer Refine Detection Kit was used according to the manufacturer’s instructions. ImageJ software (National Institute of Health, Bethesda, MD, USA) was used to quantify the amount of SP expression after immunohistochemical staining.

### 4.10. Statistical Analysis

Data were analyzed using the GraphPad Prism 6 software and are expressed as mean ± standard deviation. When the means of the two groups were compared, a Mann–Whitney *U* test was used. When the means of two variables (e.g., induction and concentration) of more than two groups were compared, a Kruskal-Wallis test with Tukey’s post-hoc test was used. Results were considered significant when *p* < 0.05.

## 5. Conclusions

Our analyses indicate that long-term exposure to SP is one of the causes of tendinopathy. This study protocol will be beneficial for use in new animal models of tendinopathy and ultimately aid better understanding of the condition. Future research is warranted to develop novel therapeutics targeting SP.

## Figures and Tables

**Table 1:** Animal groups and treatment.

Group (*n*)	Treatment	Duration	Frequency	Dose
Negative Control	PBS injection	2 weeks	EOD *	20 µL
Positive Control	Running	7 weeks	1 h/day	
SP	SP ^†^ injection	2 weeks	EOD	20 µL
SP + Run	SP injection + Running	2 weeks	EOD + 1 h/day	20 µL

* Every other day, ^†^ Substance P.

## Supplementary Materials

The following are available online at https://www.mdpi.com/1422-0067/21/22/8633/s1, Table S1: Movin score.

## Abbreviations

SP	Substance P
IL-6	Interleukin-6
COL3	Collagen type 3
COL1	Collagen type 1
PBS	Phosphate-buffered saline
qRT-PCR	Quantitative real-time polymerase chain reaction
ELISA	Enzyme-linked immunosorbent assay
ICC	Immunocytochemistry
TNMD	Tenomodulin
cDNA	Complementary DNA
H and E	Hematoxylin and eosin
