# Supplementary material for: Sustained Exposure of Substance P Causes Tendinopathy
Source: Int J Mol Sci. 2020 Nov 16;21(22):8633. doi: 10.3390/ijms21228633 (PMC7709031; doi:10.3390/ijms21228633)
Supplement: Supplementary file 1 [file ijms-21-08633-s001.pdf]

Supplementary data.

Supplementary Table S1. Movin score

Variables	Grade			
	1 (Normal)	2 (Slightly abnormal)	3 (Abnormal)	4 (Markedly abnormal)
**Fiber structure**	Continue, long fiber	Slightly fragmented	Moderately fragmented	Severely fragmented
**Fiber arrangement**	Compacted and parallel	Slightly loose and wavy	Moderately loose, wavy and cross to each other	No identifiable pattern
**Rounding of the nuclei**	Long spindle shape cells	Slightly rounding	Moderately rounding	Severely rounding
**Regional variations in cellularity**	Normal pattern	Slightly increase	Moderately increase	Severely increase
**Increased vascularity**	< 10%	10 - 20%	20 - 30%	> 30%
**Decreased collagen stainability**	< 10%	10 - 20%	20 - 30%	> 30%
**Hyalinization**	< 10%	10 - 20%	20 - 30%	> 30%
**Glycosaminoglycan content**	< 10%	10 - 20%	20 - 30%	> 30%
